# Somatostatin receptor mediated targeting of acute myeloid leukemia by photodynamic metal complexes for light induced apoptosis

**DOI:** 10.1038/s41598-019-57172-6

**Published:** 2020-01-15

**Authors:** Naidu M. Vegi, Sabyasachi Chakrabortty, Maksymilian M. Zegota, Seah Ling Kuan, Anne Stumper, Vijay P. S. Rawat, Stefanie Sieste, Christian Buske, Sven Rau, Tanja Weil, Michaela Feuring-Buske

**Affiliations:** 1 Institute of Experimental Cancer Research, Comprehensive Cancer Centre, University Hospital Ulm, D-89081 Ulm, Germany; 2 Department of Chemistry, SRM University, AP – Amaravati, Andhra Pradesh, 522502 India; 3 Max Planck Institute for Polymer Research, D-55128 Mainz, Germany; 4 Institute of Inorganic Chemistry I, Ulm University, D-89081 Ulm, Germany; 5 Department of Internal Medicine III, University Hospital Ulm, D-89081 Ulm, Germany

**Keywords:** Leukaemia, Drug delivery

## Abstract

Acute myeloid leukemia (AML) is characterized by relapse and treatment resistance in a major fraction of patients, underlining the need of innovative AML targeting therapies. Here we analysed the therapeutic potential of an innovative biohybrid consisting of the tumor-associated peptide somatostatin and the photosensitizer ruthenium in AML cell lines and primary AML patient samples. Selective toxicity was analyzed by using CD34 enriched cord blood cells as control. Treatment of OCI AML3, HL60 and THP1 resulted in a 92, and 99 and 97% decrease in clonogenic growth compared to the controls. Primary AML cells demonstrated a major response with a 74 to 99% reduction in clonogenicity in 5 of 6 patient samples. In contrast, treatment of CD34^+^ CB cells resulted in substantially less reduction in colony numbers. Subcellular localization assays of RU-SST in OCI-AML3 cells confirmed strong co-localization of RU-SST in the lysosomes compared to the other cellular organelles. Our data demonstrate that conjugation of a Ruthenium complex with somatostatin is efficiently eradicating LSC candidates of patients with AML. This indicates that receptor mediated lysosomal accumulation of photodynamic metal complexes is a highly attractive approach for targeting AML cells.

## Introduction

Acute myeloid leukemia (AML) is initiated and propagated by cancer stem cells. Although these leukemic stem cells (LSCs) initially respond to chemotherapy, most patients relapse and die from the disease^1^. One explanation is that current therapies only eliminate the tumor bulk which lacks leukemic stem cell properties whereas the LSCs are relatively insensitive. Growing knowledge of the molecular landscape of AML has led to clinical testing of new drugs against driver mutations as well as antibody-based therapies against cell surface proteins with partly disappointing results^2–5^. Thus, for the majority of patients there is therapeutic standstill and the urgent need for innovative treatment approaches. Tumor-associated peptides provide attractive characteristics for AML-targeted strategies such as easy availability, convenient purification and storage. In addition, they are less immunogenic, have a high tissue penetration and a high affinity to cellular biomarkers. They provide a rapid clearance from the body and are excellent candidates for straight forward conjugation strategies. It has been shown that somatostatin receptors (SSTR) are expressed on leukemias such as T-cell leukemia and AML^6,7^. Using a somatostatin radiobinding assay it was demonstrated that around 12.5% of AML cases express somatostatin receptors^6^. Previously, it has been shown that primary AML progenitor cells, characterized by the co-expression of CD34 and CD117 express the somatostatin receptor (SSTR) subtype 2 and that the expression of the SSTR2 receptor was not restricted to the immature CD34^+^CD117^+^ compartment, but also detected on more differentiated AML blasts. Using a transwell migration assay, it was demonstrated that the migration of AML cells towards a gradient of the synthetic analogue of somatostatin octreotide, correlated with the expression of SSTR2^8^. indicating that somatostatin might influence spreading of AML cells^8^. Based on these findings, somatostatin receptors might be a potential interesting target for the treatment of AML. In the current approach we analysed the AML targeting potential of an innovative biohybrid consisting of the tumor-associated peptide somatostatin and the photosensitizer ruthenium using molecularly and cytogenetically fully annotated primary AML patient samples as targets.

Our own previous results using lung cancer cell lines demonstrated that this new biohybrid is highly selective and potent: cellular uptake is mediated by clathrin-dependent endocytosis and followed by efficient generation of singlet oxygen upon irradiation with negligible dark toxicity.^9^. These results were now translated into a pre-clinical setting mimicking the potential application of such a construct in the context of *ex vivo* purging of autologous bone marrow (BM) transplants in AML.

## Materials and Methods

### Cell culture, AML cell lines and primary samples

Quantitiative real-time PCR analyses of the expression of SSTR2 as well as functional testing of the RU-SST compound were performed on the following leukemic cell lines: OCI-AML3 (OA3), THP-1, HL60, MonoMac6 (MM6), K562, KASUMI, MV4-11, Nalm6, NB4 (all DSMZ, Braunschweig, Germany). Cells were cultured in RPMI 1640 medium with 10% FBS (K562, HL60, THP1, Nalm6, MV4-11, and MM6) or 20% FBS (OCI-AML3) supplemented with 1% penicillin-streptomycin.

Mononuclear cells were isolated from bone marrow (BM) or peripheral blood (PB) from patients with primary diagnosed AML, analyzed for their SSTR2 expression (n = 13) and cultured with the RU-SST bioconjugate to test the toxicity of the compound (n = 6). All patient samples were investigated by cytomorphological, cytogenetic and molecular analyses after written informed consent as described^10^. Diagnosis was made according to the French-American-British criteria and the World Health Organization classification (Table 1)^11,12^. The study was approved by the ethic committee of the University of Ulm. in accordance with the ethical principles of the declaration of Helsinki (http://www.wma.net/en/30publications/10policies/b3/index.html). Leukemic cells were thawed and kept in culture with culture conditions as mentioned previously^13^.

**Table 1:** Patients’ characteristics of samples incubated with RU-SST and RU-Alkyne.

Patient no.	Gender	Age (years)	Karyotype	Other relevant markers
1	F	33	46XX[20]	NPM1 mut
2	F	61	46XX[13]	NPM1 mut
3	F	75	46,XX [20]	None
4	M	72	47,XY [14]	None
5	F	63	46,XX[20]	NPM1 mut
6	F	45	45,XX,-7[20]	FLT3-ITD
7	M	22	46,XY,del(7)(q32q36), inv(16)(p13.1q22)[19] 46,XY[1]	None
8	F	87	47,XX, + 13[9] 46,XX[11]	FLT3-TKD
9	M	23	46,XY[20]	FLT3-ITD
10	F	43	46XX[23]	NPM1 mut, FLT3-ITD
11	M	60	47,XY + 8[20]	None
12	F	43	46,XX[20]	NPM1 mut, FLT3-ITD
13	F	41	n.e.	FLT3-ITD

Cells from cord blood (CB) were enriched for CD34^+^ using the human CD34 Micro Bead Kit ultrapure (Miltenyi Biotec, Auburn, CA, USA) according to the manufacturer instructions.

### Annexin V staining

Apoptosis was measured by Annexin V staining using the Annexin V apoptosis detection kit (BD Biosciences 2350 Qume Drive, San Jose, CA 95131). Experiments were performed according to the manufacturer’s protocol. In brief, OCI-AML3, MM6 and THP-1 cells were incubated with RU-SST as described in the Materials and Methods part. Subsequently, cells were washed and incubated with 7-AAD and Annexin V for 15 min. at room temperature. Samples stained with Annexin V or 7-AAD alone were taken as controls. The analysis was performed on a Fortessa^TM^ flow cytometer (Becton Dickinson, CA, USA).

### CFC assay

To evaluate the toxicity of the bioconjugate on clonogenic progenitor cells, AML cell lines as well primary human cells were incubated with the compound for 4 hours. Subsequently, cells were washed and cell counts were determined via trypan blue exclusion. Colony forming cell unit (CFC) assays were performed as described previously^14^. In all cases cell numbers placed in methylcellulose were calculated according to the initial cell number at the start of the experiment, not taking into account any cell loss during the incubation time. 1000 cells (OCI-AML3 and HL60) and 500 cells (THP1) were plated per dish (methocult H4330). For primary patient samples, 10,000 cells were plated per dish in methylcellulose H4330 (Stem Cell Technologies, Vancouver, BC, Canada) and supplemented with cytokines. For the CD34^+^ enriched CB cells, 300 cells were plated per dish in methocult H4434 (Stem Cell Technologies, Vancouver, BC, Canada). Each experiment was performed in duplicates and values mentioned are Mean ± SEM.

### Determination of reactive oxygen species

OCI-AML3 and CD34^+^ CB cells were placed on Retronectin (r-fibronectin) (Takara; CH-296 cat#T100A) overnight. CD34^+^ CB were kept in culture in IMDM medium containing BIT (Stem Cell Technologies, Vancouver, BC, Canada) and supplemented with a cytokine cocktail containing 100 ng/mL Flt-3 ligand, 100 ng/mL steel factor, 20 ng/mL interleukin-3, 20 ng/mL IL-6, and 20 ng/mL granulocyte colony-stimulating factor (G-CSF) as described previously^15^. Cells were washed gently with PBS and incubated with fresh medium containing RU-SST for 4 hours in duplicates. Subsequently, cells were washed again and half of the cells was either exposed for 6 minutes to light or kept in the dark (Dark control)^9^. Cells were washed twice with PBS. Pre-warmed staining solution containing Cellular Reactive Oxygen Species Detection Assay Kit was added (Deep Red Fluorescence) (Abcam, Cambridge, UK). The cells were incubated for 30 min at 37 °C. After staining cells were carefully washed and replaced with HyCloneTM DMEM medium (VWR, Darmstadt, Germany) and proceeded for observation under a confocal microscope or for flow cytometric analysis. The live cell imaging was performed using a LSM 710 laser scanning confocal microscope system (Zeiss, Germany) coupled to an XL-LSM 710 S incubator and equipped with a 63x oil immersion objective. The acquired images were processed with ZEN 2011 software (Zeiss, Oberkochen, Germany)^9^. The corrected total cell fluorescence (CTCF) was calculated as previously described^16–18^ using the ImageJ v1.5 software (NIH, USA).

For flow cytometric analysis of ROS cells were further treated with propidium iodide in order to determine dead cells and analyzed on a FACS Fortessa^TM^ (Becton Dickinson). PI negative (alive) cells were gated and analyzed for the expression of ROS.

### Statistical analyses

Statistical analysis was performed using the Mann-Whitney test for independent samples. Flow cytometry data were analyzed with FlowJo™ (Becton Dickinson). Differences with *p* values less than 0.05 were considered to be statistically significant (*p < 0.05; **p < 0.01; ***p < 0.001; ****p < 0.0001). Values mentioned are Mean ± SEM. GraphPad PRISM® 6 (Version 06.01; La Jolla, California, USA) was used for the analyses and figures. Correlation coefficients were calculated using Microsoft Excel 2010.

## Results

### Expression of somatostatin receptors in cytogenetic subgroups of AML compared to normal progenitor cells

To investigate the potential role of somatostatin receptors as targets for anti-leukemic therapy, we analyzed AML cell lines representing various cytogenetic subgroups for the expression of somatostatin receptors by qRT-PCR. SSTR2 expression was detected in all cell lines tested, with the highest expression in THP-1 (Supplemental Fig. 1). In addition, we analyzed published data using RNA-Seq^19^ (GSE49642) from 43 primary AML patient samples. We observed that SSTR2 and to a lesser extent SSTR3 were expressed in a part of AML patient samples (Supplemental Fig. 2A). Those patient samples which showed the highest expression had a normal karyotype together with a mutation of the nucleophosmin 1 gene^19^. SSTR2 expression was also present in other subtypes of AML as demonstrated in the microarray analysis of various AML data sets including the TCGA and MILE data (Supplemental Fig. 2B). In contrast, SSTR2 was not or only low expressed in HSC and dimly expressed in MPP, BC and CMP depending on the probe set (Supplemental Fig. 2B). To evaluate whether SSTRs would also be expressed on normal early hematopoietic progenitor cells, we further examined published RNASeq data from sorted subpopulations from CB^20^. Among all somatostatin receptors it was SSTR2 which was expressed mainly in the megakaryocyte erythroid progenitor cells and seemed to be significantly lower expressed especially on the most primitive HSC population (Supplemental Fig. 2C). RNA sequencing experiments showed similar results with a high expression of SSTR2 in CD34 positive hematopoietic stem cells as well as proerythroblasts^21^ (Supplemental Fig. 2D).

### Stability of the RU-SST bioconjugate

The ruthenium complex (RU) and the peptide hormone somatostatin (SST) were conjugated as described previously in order to combine the LSC selectivity of somatostatin with the potent photosensitizer ruthenium utilizing CLICK chemistry approaches^9^. A lysine residue is located within the SST receptor binding domain. Therefore, non-specific lysine modifications are not applicable for the conjugation of SST. However, N-terminal modification could be applied via solid phase synthesis to maintain the binding properties of the SST^9^.

The stability of RU-SST was analyzed by liquid mass spectrometry (LC-MS) using similar culture conditions as in the cell culture experiments. The LC-MS study showed that the amount of RU-SST present in 10% fetal calf serum (FCS) in PBS remains consistent up to 8 h. Furthermore, there was no additional peak observed in the liquid chromatogram at 254 nm, which would indicate peptide fragmentation (Supplemental Fig. 3A–C). Taken together, these data indicated that RU-SST is stable up to 8 h in FCS.

### Uptake and efficiency of RU-SST

**Figure 1:**
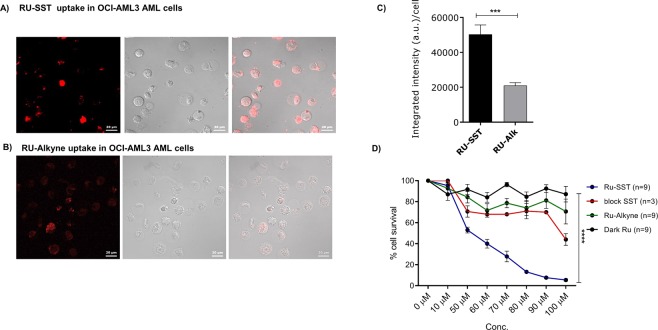
Uptake and IC50 studies of RU complex: confocal images of OCI-AML3 cells incubated with RU-SST (50 μm) (**A**) and RU-Alkyne control (RU-Alk) (100μm) (**B**) for 4 hrs. The laser intensity was measured for alexaflour (430 nm). Uptake characteristics with values of the integrated intensity (a.u.) per cell (**C**). Bar graphs indicate the average integrated intensity per cell in both experimental arms. Cells analysed for RU-SST (n = 45) and RU-Alk (n = 44). Data are represented as mean ± SEM. (**D**) IC50 was calculated in HL60 AML cell lines. Percent cell survival was measured by trypan blue exclusion after 4 h incubation with RU-Alk (control), RU-SST (irradiated) compared to RU-SST in the absence of light (dark RU). Experiments performed for dark RU-SST (n = 6); RU-Alk (n = 3), for RU-SST (n = 3). Data are represented as mean ± SEM.

To demonstrate the superior cellular uptake and efficiency of RU-SST within leukemic cells we chose the OCI-AML 3 and HL60 cell lines. OCI-AML3 (OA3) cells were incubated with RU-SST or the unconjugated RU-Alkyne control for 4 hours and analyzed using laser scanning confocal microscopy^9^. SSTR2 expression was confirmed by RT-PCR and western blot (data not shown). Uptake studies were performed in OA3 using a laser excitation at 458 nm which corresponds to the Metal to Ligand Charge Transfer (MLCT) absorbance of the Ruthenium metal complex. Emission images ranging from 580–707 nm were recorded^9^. We could clearly observe a high uptake of RU-SST in OA3 cells with an average integrated intensity of 40302 A.U./cell (range 111030-3932 A.U./cell), demonstrating that the biohybrid RU-SST was transported adequately into the cells. There was a significantly higher emission intensity inside the cells compared to the control experiment using the unconjugated RU-Alkyne (average integrated intensity of 12898 A.U./cell, p < 0.0001, Mann-Whitney test) (Fig. 1A–C).

To determine the effective concentration of RU-SST and to compare it with the unconjugated control, HL60 cells were incubated with increasing concentrations of the compound and RU-Alkyne, respectively, for 4 hrs in the dark and subsequently exposed to light. RU-SST was highly toxic to HL60 cells with an IC50 value of 47.4 ± 3 µM after light irradiation (Fig. 1D). The IC50 was not reached with up to 100 µM for RU-SST or the model complex in the dark. RU-SST displayed a phototoxicity index (PI) of >2.1 (PI is obtained by dividing dark and light IC50 values). RU-SST (IC50 = 47.4 ± 3 µM) was also more effective when compared to the unconjugated RU-Alkyne complex (IC50 > 100 µM), pointing to a superior cellular uptake and thereby leading to a greater potential of RU-SST for photodynamic therapy (PDT). Cellular uptake of RU-SST could be blocked upon addition of an SSTR 2 specific antibody further verifying ligand mediated SST functionalization (Fig. 1D).

### RU-SST effectively impairs AML growth

To measure the effect of RU-SST on leukemic clonogenic progenitor cells we placed cell lines and primary patient samples in methylcellulose assays.

**Figure 2:**
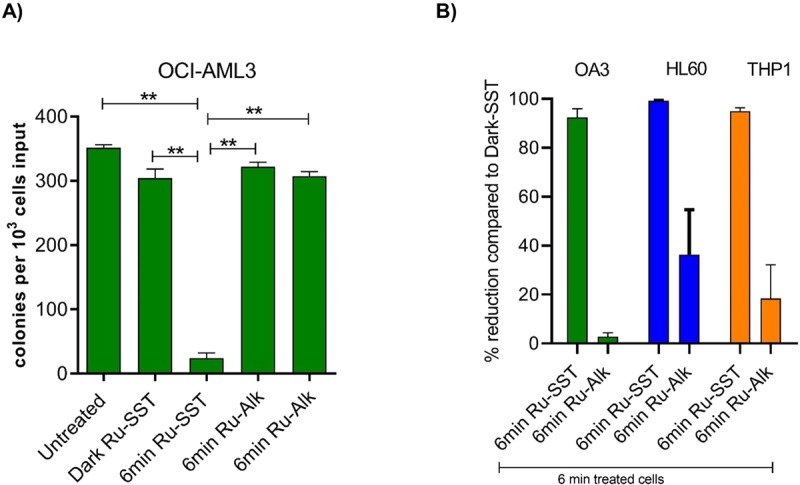
Determination of clonogenic potential of AML cell lines with RU-complexes: (**A**) Bar graph shows the number of colonies after day 14 of plating cells on methylcellulose in OCI-AML3 AML cells. Significance was calculated using the Mann-Whitney test (** < 0.01). (**B**) Percent reduction in the number of colonies compared to dark control in OCI-AML3, HL60 and THP-1 AML cells. The bars represent mean ± SEM (n = 3).

We could detect a significant decrease in colony growth in the OCI AML3 cell line with a 92% reduction of colony growth when the cells were incubated with RU-SST and exposed to light compared to the cells treated with RU-SST and not exposed to light (Fig. 2A). Interestingly, the incubation of the cells with RU-SST and without light exposure did not cause a significant reduction in colony growth when compared to an untreated control. Furthermore, there was no comparable reduction in colony growth when the cells were treated with the unconjugated RU-Alkyne control and exposed or not exposed to light. A similar response to RU-SST with exposure to light was also observed in the HL60 and THP1 cell lines with a 99% and 97.66% reduction of colony growth respectively, compared to the RU-SST dark control (Fig. 2B) (p < 0.002; Mann-Whitney test). Treatment of these cell lines with increasing concentrations of the somatostatin analogue octreotide did not affect cell proliferation (Supplemental Fig. 4). Toxicity of the compound was due to the induction of apoptosis as demonstrated for the THP1, Mono Mac 6 and HL60 cell lines (Supplemental Fig. 5).

To test the efficacy in primary AML samples, we chose patient samples from which we could previously demonstrate that they were able to form colonies in methylcellulose assays, representing a small subset of AML genotypes (data not shown). Quantitative expression analysis demonstrated that these patients (n = 6) showed a variable expression of SSTR2 receptors (Supplemental Fig. 6). After treatment, CFC counts demonstrated an average reduction of colony numbers of 89.5% (±4.0% SEM) in 5 out of 6 samples and 44.79% reduction in 1 out of 6 samples analysed at day 14 in comparison to samples treated but not exposed to light (Dark RU-SST).). In contrast, the unconjugated control (RU-Alkyne) did not show a significant reduction in CFC numbers with an average reduction of 17.6 (±7.03 SEM) (dark Ru-Alkyne) and 31.4 (±8.97% SEM) RU-Alkyne with exposure to light, respectively (Table 2).

**Table 2:** Toxicity of Ru-SST on clonogenic progenitor cells.

CFC/10^4^ cells (% reduction)				
	control	6 min RU-SST	Dark RU-Alk	6 min RU-Alk
AML#1	100	26 (74)	153 (0)	78 (22)
AML#2	252	139 (44.7)	247 (2.0)	208 (17.7)
AML#3	137	1 (99.2)	187 (0)	211 (0)
AML#4	47	5 (89.3)	29 (38.2)	24 (48.9)
AML#5	118	4 (96.6)	72 (38.9)	69 (41.5)
AML#6	70	8 (88.5)	36(48.5)	29 (58.7)

*control = dark RU-SST.

### Uptake and toxicity in cord blood cells

**Figure 3:**
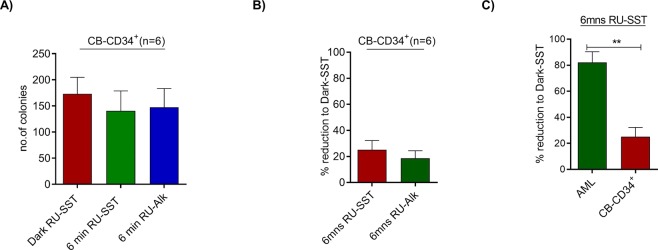
Effect of RU-complexes on clonogenicity of normal CD34^+^ cells: cells from four CD34^+^ enriched cord blood (CB) samples (n = 6) were tested for the effect of both RUSST and RU-Alkyne compounds. The bar graphs represent the average colony numbers of each experimental arm performed in duplicates. Percent reduction was determined based on the colony numbers generated from CB cells treated with RU-SST and exposed to light compared to dark RU-SST. C) Bar graphs show the percent decrease in CFC growth of AML samples compared to normal CB CD34^+^ cells. Significance calculated by Mann-Whitney test ** < 0.01.

To evaluate, whether the effect of the compound was selective for AML stem and progenitor cells, we analysed the toxicity of the compound for CD34^+^ enriched CB cells (n = 6). Uptake studies were performed for both conjugates prior to proceeding to the experiments. We could observe a high uptake of RU-SST in CB cells with an average integrated intensity of 35521.14 ± 5541 SEM A.U./cell (range 59221 to 12184 A.U./cell), demonstrating that the biohybrid RU-SST was transported efficiently into the CB cells despite lower SSTR2 expression on HSCs. The emission intensity inside the cells was significantly higher compared to the control experiment using the unconjugated RU-Alkyne (average integrated intensity of 3409.25 ± 366.2 SEM A.U./cell, p < 0.0001, Mann-Whitney test) (Supplemental Fig. 7A,B). To test the toxicity of the compound on colony growth, we treated CB cells with RU-SST and the unconjugated RU-Alkyne as described. We observed a mean reduction of 25.4% (±6.82 SEM%) in colony numbers when the CB cells were treated with RU-SST and exposed to light with an average colony number of 140.6 CFC per 300 input cells (±37.9 SEM) compared to 173.2 colonies per 300 input cells in the dark control (±31.3 SEM) (Fig. 3A). The unconjugated RU-Alkyne only showed a minor toxicity on normal CB cells with a reduction of 18.59% (±4.88% SEM) in colony numbers compared to dark RU-SST (Fig. 3B). These data point to a superior toxicity of RU-SST on AML samples compared to healthy CD34^+^ stem cell candidates (Fig. 3C).

### Lysosomal localization and generation of reactive oxygen

**Figure 4:**
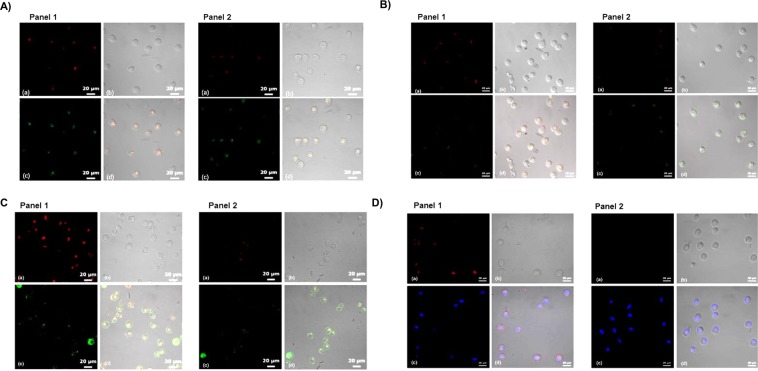
Evaluation of localization of the RU complexes: (**A**–**D**): (**A**) Confocal microscopy images of OCI-AML3 cells incubated with RU-SST (Panel 1) and RU-Alkyne (Panel 2) and treated with organelle trackers for (**A**) lysosomes, (**B**) mitochondria, (**C**) membrane and (**D**) nuclei. The panels of each figure show (a) RU-SST emission, (b) corresponding bright field images, (c) emission from the organelle trackers, and (d) overlay of all three images.

**Figure 5:**
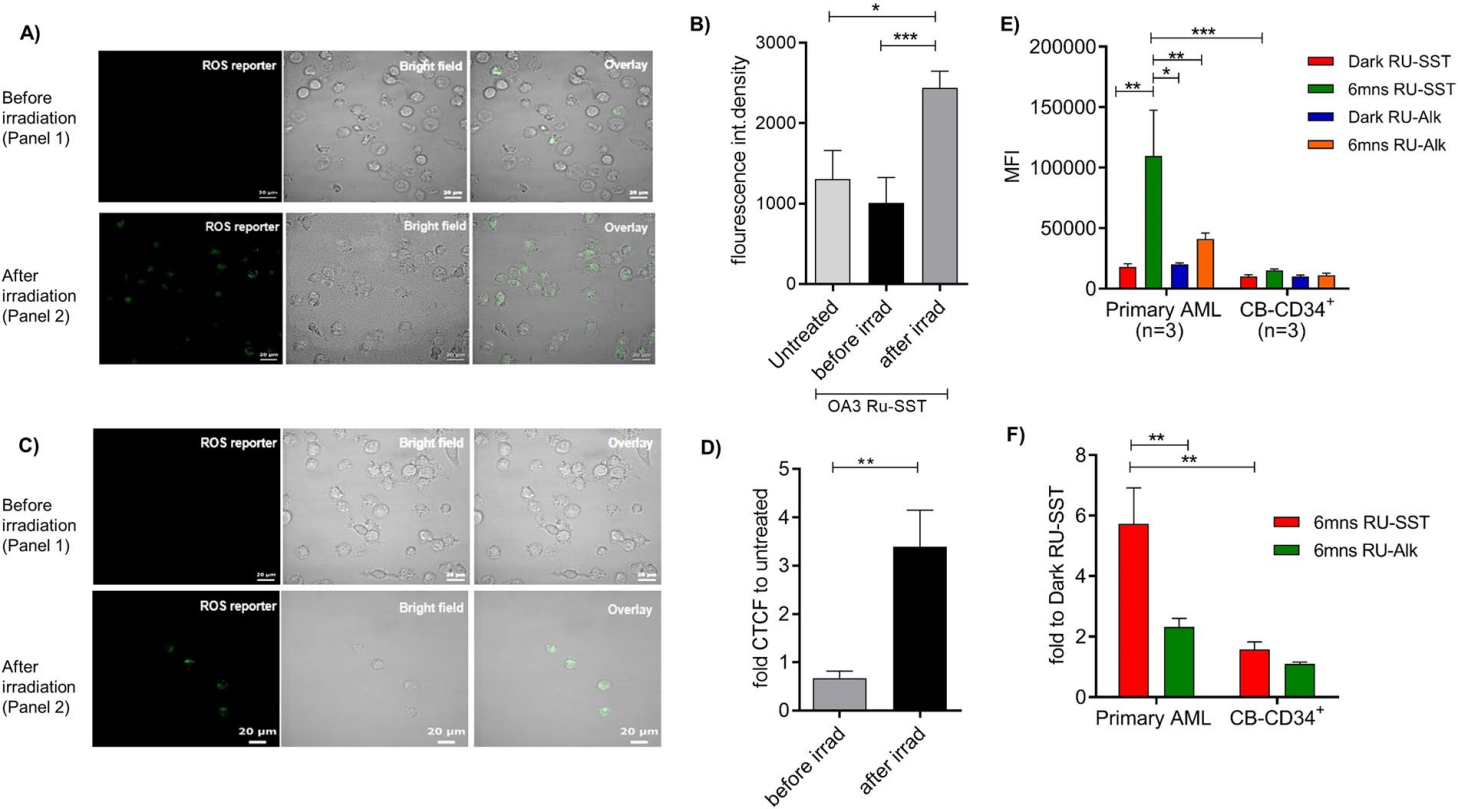
Determination of reactive oxygen species (ROS) levels (**A,C**) Confocal microscopy images of OCI-AML3 cells incubated with RU-SST (**A**) and RU-Alkyne (**C**) and treated with ROS reporter and kept in the dark (Panel 1) or exposed to light (Panel 2). (a) ROS reporter, (b) corresponding bright field image, (c) overlay. (**B**) Bar graph indicates the fluorescence intensity corresponding to A) for untreated OCI-AML3 cells, after treatment with RU-SST in the dark as well as after exposure to light (irradiatíon). Significance calculated by Mann-Whitney test *** < 0.001 and * < 0.05. (**D**) Bar gaph indicates the fold CTCF (The corrected total cell fluorescence) of ROS reporter before and after irradiation compared to untreated cells. (**E**) Bar graph indicated the Mean fluorescence intensity (MFI) of ROS measurement in primary AML (n = 3) and nCB-CD34^+^ (n = 3) cells. (**F**) Bar graph indicates the fold increase of MFI after irradiation with RU-SST compared to Dark control in primary AML samples and were compared to the fold reduction in nCB-CD34^+^ cells (n = 3). Significance was calculated by 2way ANOVA with *p < 0.05, **p < 0.01.

To evaluate, whether the differential toxicity of RU-SST was due to a different uptake or subcellular localization we used different subcellular organelle dyes and laser scanning confocal microscopy. In OCI-AML3 cells, there was a clear and very strong co-localization of RU-SST in the lysosomes (Pearson’s coefficient 0.94, Fig. 4A, strong to moderate co-localization in membranes (Pearson’s coefficient 0.70, Fig. 4B) and mitochondria (Pearson’s coefficient 0.60, Fig. 4C), and a poor co-localization in the nucleus (Pearson’s coefficient 0.35, Fig. 4D). In comparison, CD34^+^ CB cells showed almost no significant uptake of the bioconjugate in the same cellular compartments tested (Supplemental Fig. 8A–D), suggesting that the lysosomes and mitochondria may be the intracellular targets for photodynamic killing of leukemic cells mediated by RU-SST in AML cells. Experiments measuring ROS generation demonstrated that intracellular localization of RU-SST induced high levels of ROS in OCI-AML3 cells after exposure to light compared to the treated dark control cells (Fig. 5A,B,D) as well as to the RU-Alkyne control (Fig. 5C). Flow cytometric analysis of reactive oxygen species (ROS) in primary AML samples (n = 3) demonstrated a significant higher production of ROS in primary AML cells compared to CD34 positive CB cells. In detail, the mean fluorescence intensity was 5.7 (±1.19 SEM) fold higher in the RU-SST arm compared to the RU-SST dark control and 7.4 (±2.9 SEM) higher when compared to the RU-SST treated CD34^+^ CB control cells (p < 0.05) (Fig. 5E,F). These data indicate that the differential generation of reactive oxygen species induced by RU-SST in primary AML samples versus the CB progenitor cells is responsible for the pronounced toxicity of the compound in patient samples.

## Discussion

Chemotherapy treatment of patients with newly diagnosed AML consists of a remission induction followed by a post-remission therapy (PRT). Remission induction is applied to reduce the leukemic cell burden in a patient below the level of morphological detectability. Post-remission therapy is needed because 70–80% of patients who initially respond to the induction therapy ultimately relapse and die without subsequent additional treatment due to persistent minimal residual disease^1^.

Autologous hematopoietic cell transplantation (auto-HSCT) is one option for a post-remission therapy: previous studies could show its beneficial effect in patients with good risk cytogenetics^22,23^. Superior outcome has been demonstrated in patients with CBF^24^ NPM mutated^25^ and CEBPA double mutated (CEBPA dm)^26^ AML. Potential disadvantages of auto-HSCT consists of the lack of a graft versus leukemia effect and a contamination of the graft with leukemic stem and progenitor cells, which might contribute to relapse^27^.

Purging strategies have been developed and tested over the last decades using physical systems, cytotoxic therapies, immunological *in vitro* and *in vivo* techniques, molecular and genetic methods *ex vivo* cultivation as well as cytokine mediated activation of immune effector cells with varying success^28–32^.

Limitations of these strategies were toxicity on normal hematopoietic stem cells, lack of expression of targets on leukemic stem cells, requirement of long exposure time with subsequent loss of engrafting capacity and a subsequent delay in hematopoietic recovery resulting in a higher morbidity and mortality.

In the current approach, we analysed the AML targeting potential of an innovative biohybrid consisting of the tumor-associated peptide somatostatin and the photosensitizer ruthenium using molecularly and cytogenetically fully annotated primary AML patient samples. As ASCT is one treatment option for AML patients, photodynamic therapy (PDT) might be a beneficial approach to eliminate contaminating AML cells from an autologous graft. Furthermore, it might help to separate and examine AML cells by taking advantage of the tumor-localizing properties for fluorescence diagnosis and fluorescence activated cell sorting.

In a first step, we analysed the expression of somatostatin receptors 1–5 on leukemic cells from AML cell lines and primary AML patient samples. Mainly, SSTR2 was expressed in various myeloid leukemic cell lines as well as on leukemic cells of patients with AML. In contrast, expression of SSTR2 was negligible on HSCs from healthy donors, indicating that SSTR2 might be a potential target for therapy. Somatostatin-receptor-targeted anti-cancer therapy has been developed by conjugating various chemotherapeutic agents or radionuclides to SSTR2-preferential somatostatin analogues^33^. However, conjugation, synthesis and purification set significant limitations. In this study, we used a biohybrid consisting of somatostatin conjugated to the potent photosensitizer Ruthenium. Ruthenium(II) complexes are attractive candidates for PDT as they provide favourable features such photophysical properties, easy synthesis, flexible physical properties (i.e. charge, lipophilic properties or redox potential by coordination of the appropriate ligands), low sensitivity of photochemical properties to pH-value variations^34,35^. In addition, they are characterized by low side effects on healthy tissues, when tested *in vivo* in mouse models^36^. Therefore, ruthenium(II) complexes and their bioconjugates are particularly attractive for targeted PDT.

Treatment of OCI-AML3, HL60 and THP-1 with this biohybrid resulted in a 92%, 97.5% and 99% decrease in CFCs compared to the controls. Primary AML cells from patients with AML at diagnosis demonstrated a major response with a 74–99% reduction in CFCs in 5 of 6 and a minor response with a 45% reduction in CFCs in 1 of 4 patient samples. In contrast, treatment of CD34^+^ CB cells resulted in a substantially lower reduction in CFCs.

Subcellular localization assays of RU-SST in OCI-AML3 cells confirmed the clear co-localization of RU-SST in the lysosomes (Pearson’s coefficient 0.94) compared to a moderate to low localization in other cellular sub compartments like mitochondria and nucleus. Lysosomes are critically involved in fundamental processes such as protein secretion, endocytic receptor recycling, energy metabolism, autophagy and cell signaling^37^. It has been demonstrated that a variety of cancers use the autophagy–lysosome pathway in order to avoid cell death, immune surveillance, and deregulating metabolism^38^. Based on this, targeting of lysosomes promises to substantially impair cancer growth. Furthermore, cancer cells use lysosomes to degrade cancer drugs in an acidic environment^37^. Lysosomal targeting has been shown to be successful in leukemic stem cells through increased ROS production^39^. Targeting lysosomal enzymes can eradicate imatinib resistant CML^40^. Furthermore, it was shown, that the size of the lysosomes is significantly larger in AML stem and progenitor cells as compared to their healthy controls and that targeting of lysosomes in AML patient samples might be an attractive and novel approach for an anti-leukemic therapy^39^. Our data show that the conjugation of a Ruthenium complex with somatostatin could be highly attractive to eradicate leukemic cells of patients with AML. The lysosomal localization along with SSTR receptor mediated approach might be beneficial as it imposes a potential apoptosis route via subcellular and/or cellular targeting strategy keeping the healthy cells intact.

Analysing the effect of RU-SST on the generation of reactive oxygen species (ROS) revealed a 7.4-fold increase in ROS levels in primary AML cells compared to normal CD34^+^ cells by flow cytometry indicating that the ROS generation is one of the key mechanisms for the apoptosis mediated cell death in AML cells induced by RU-SST. Induction of reactive oxygen species (ROS) has been shown to be a major mechanism of anti-cancer drugs: this has been shown also in AML at the level of LSCs, with reducing LSC load but sparing normal HSCs. ROS induction has been described for ABT-737 and ABT-263, both Bcl-2 inhibitors^41^, Parthenolide (PTL)^42^ and dimethylaminoparthenolide (DMAPT)^43^, the vitamin A derivative Fenritinide^44^, Niclosamide^45^, Celastrol and 4-hydroxy-2-nonenal (HNE)^46,47^, 3-deazaneplanocin A (DZNep) and Mefloquin^48^.

To the end, it has to be emphasized that the effect of RU-SST might vary in different AML subtypes and that only a small subset of AML subtypes were tested in this study. However, in the current analysis the induction of reactive oxygen species by RU-SST after exposure to light together with its subcellular localization point to an effective mechanism to treat AML progenitor cells in a variety of AML genotypes.

## Supplementary information

.Supplementary information 

## Data Availability

No datasets were generated or analysed during the current study.
