# Supplementary material for: Somatostatin receptor mediated targeting of acute myeloid leukemia by photodynamic metal complexes for light induced apoptosis
Source: Sci Rep. 2020 Jan 15;10:371. doi: 10.1038/s41598-019-57172-6 (PMC6962389; doi:10.1038/s41598-019-57172-6)
Supplement: Supplementary file 1 — .Supplementary information [file 41598_2019_57172_MOESM1_ESM.pdf]

Somatostatin receptor mediated targeting of acute myeloid leukemia by photodynamic metal complexes for light induced apoptosis

Authors

Naidu M. Vegi[1], Sabyasachi Chakrabortty[2,3], Maksymilian M. Zegota[3], Seah Ling Kuan[3], Anne Stumper[4], Vijay P. S. Rawat[1], Stefanie Sieste[3,4], Christian Buske[1], Sven Rau[4], Tanja Weil[3,4], *Michaela Feuring-Buske[1,5]

Affiliations

1 Institute of Experimental Cancer Research, Comprehensive Cancer Centre, University Hospital Ulm, D-89081 Ulm, Germany

2 Department of Chemistry, SRM University, AP – Amaravati, Andhra Pradesh, 522502 India

3 Max Planck Institute for Polymer Research, D-55128 Mainz, Germany

4 Institute of Inorganic Chemistry I, Ulm University, D-89081 Ulm

5 Department of Internal Medicine III, University Hospital Ulm, D-89081 Ulm, Germany

Supplemental Figure 1

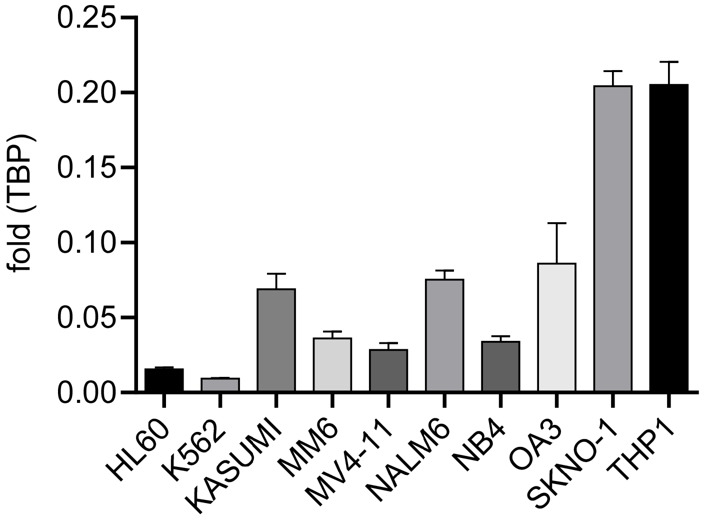

Supplemental Figure 2

A)

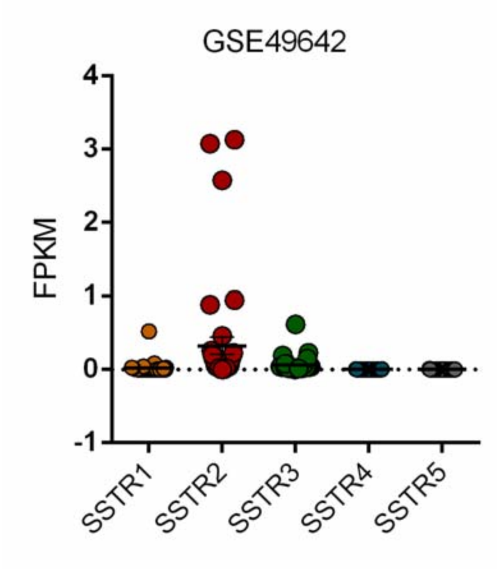

Supplemental Figure 2

B)

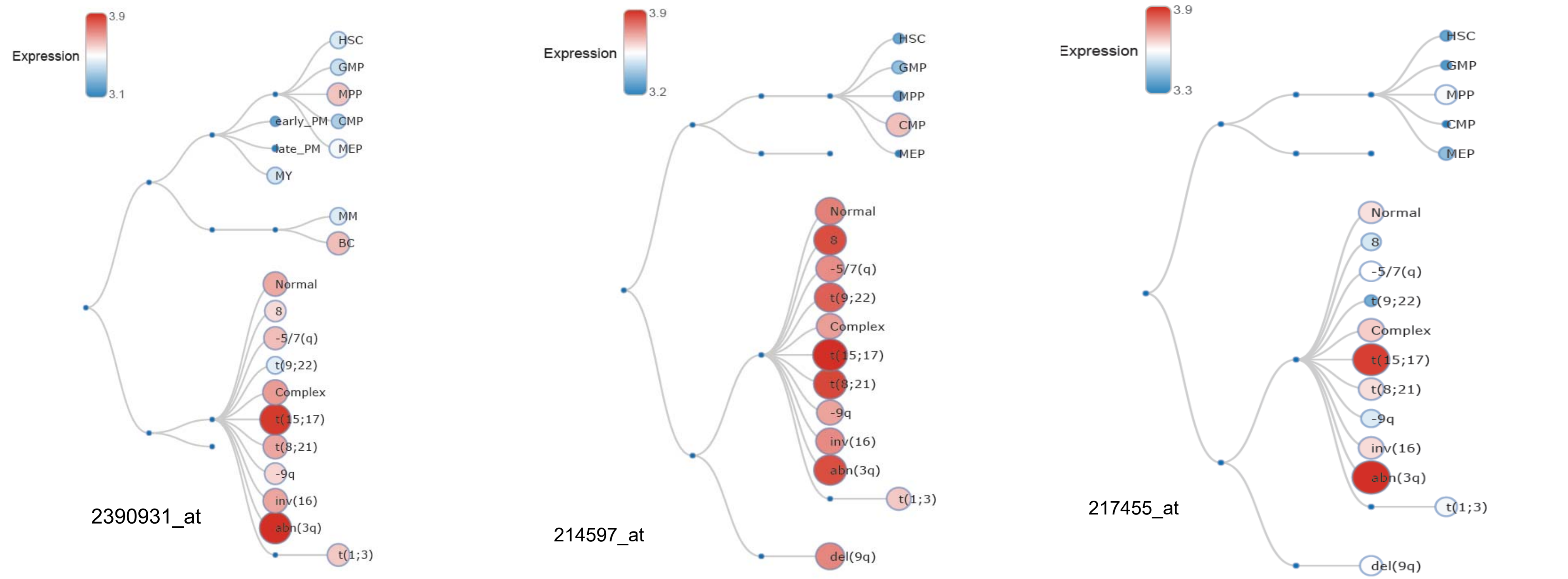

Blood pool: AML cells with normal cells

Supplemental Figure 2

C)

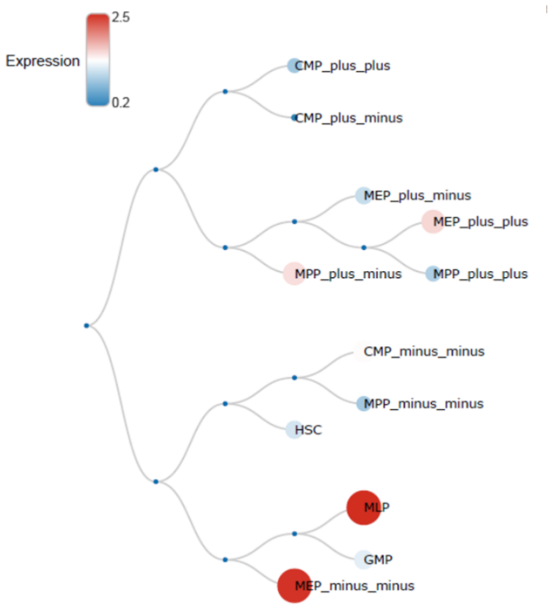

D)

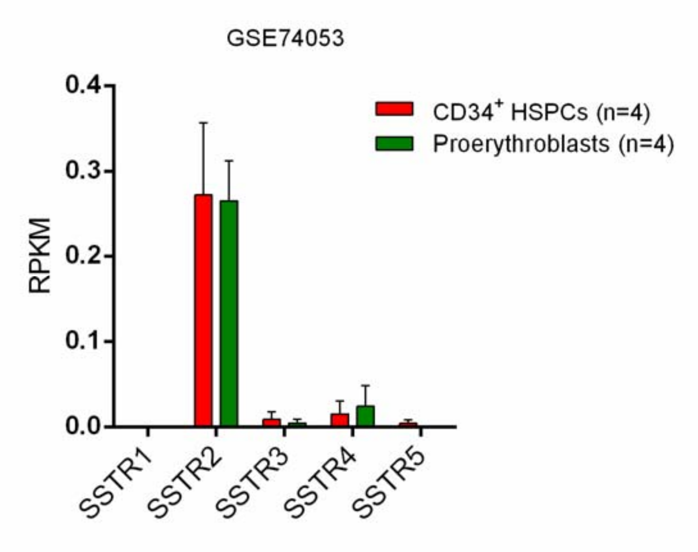

Supplemental Figure 3 A

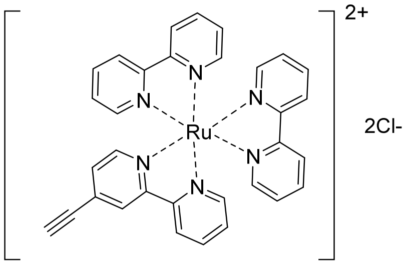

RU-Alk

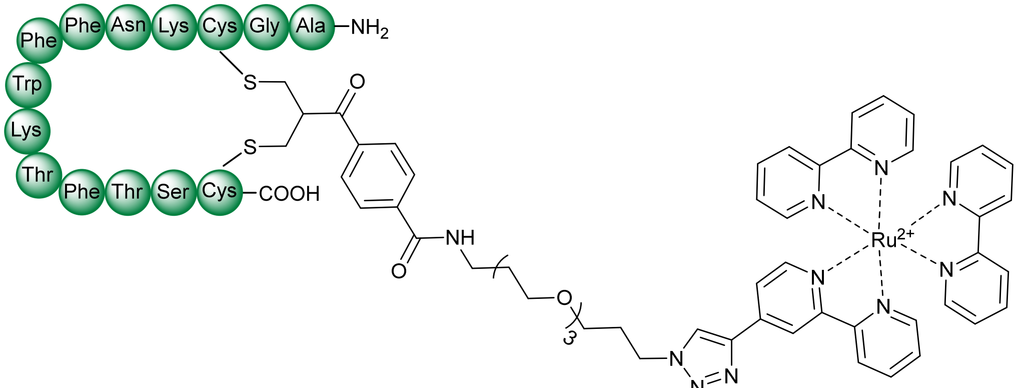

RU-SST

Supplemental Figure 3

B)

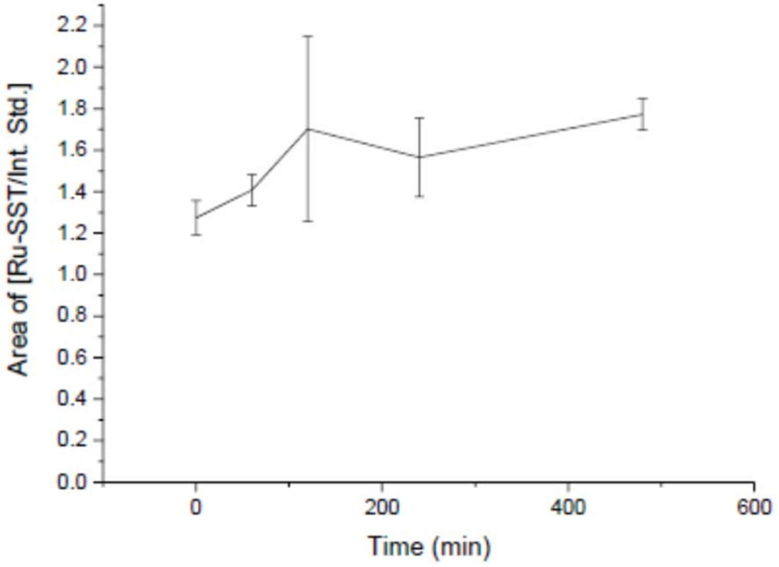

C)

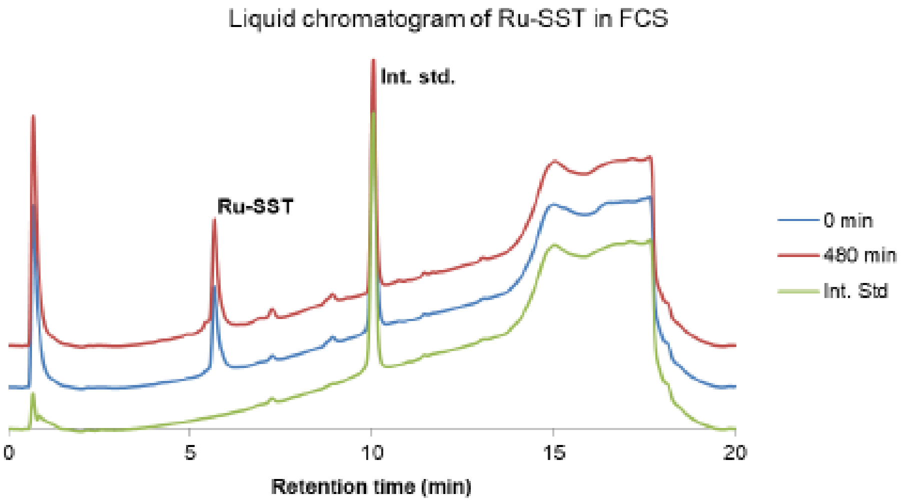

Supplemental Figure 4

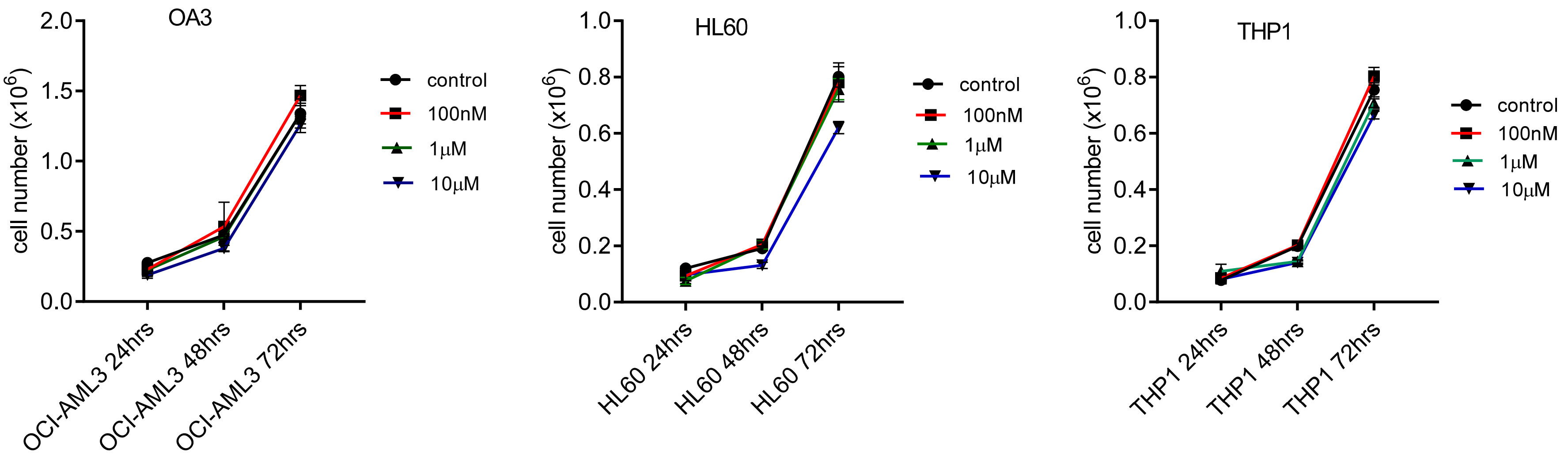

Supplemental Figure 5

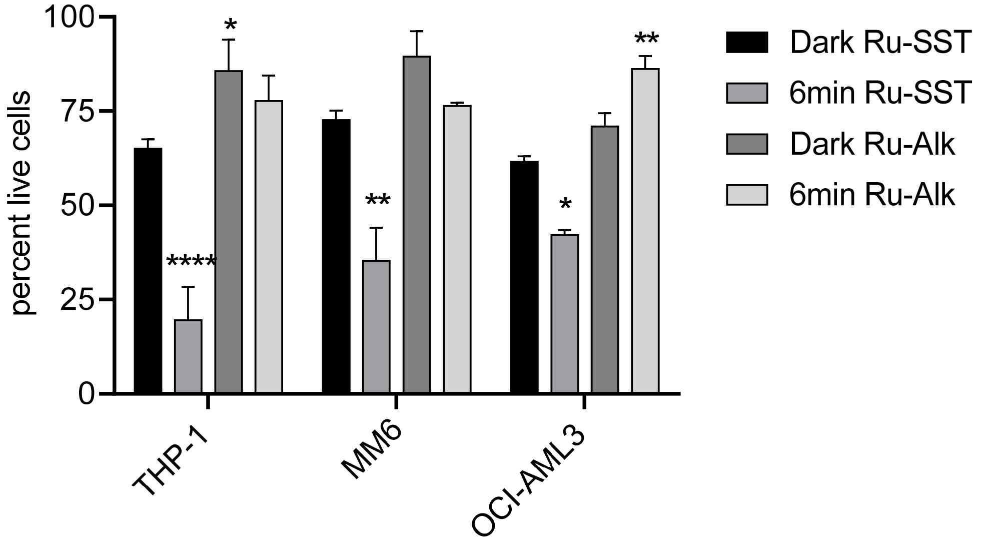

Supplemental Figure 6

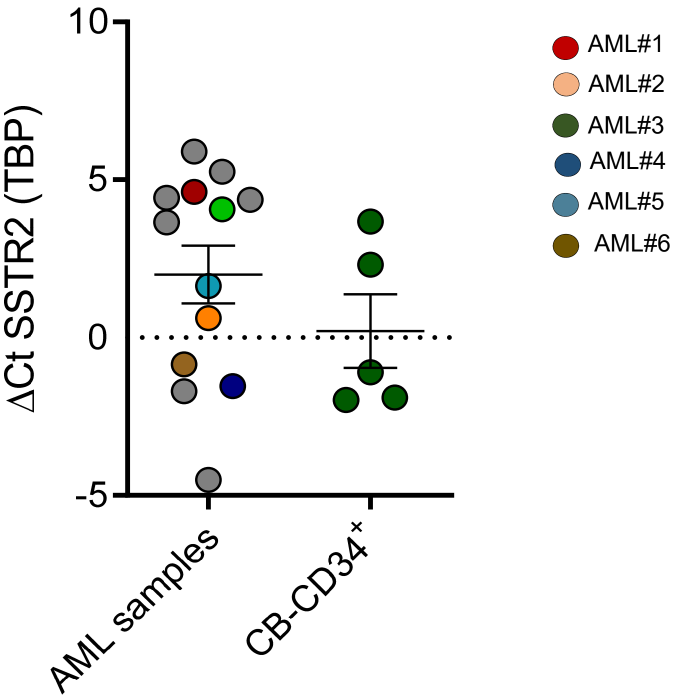

Supplemental Figure 7

A)

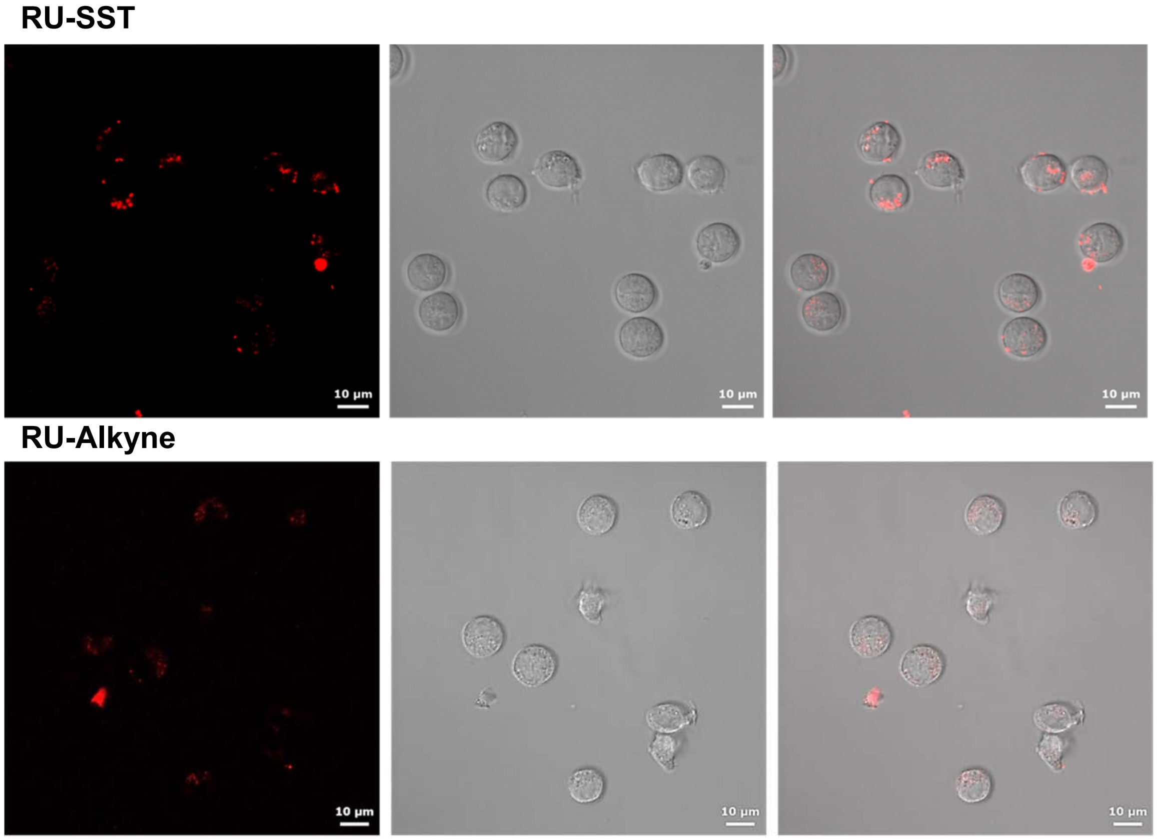

B)

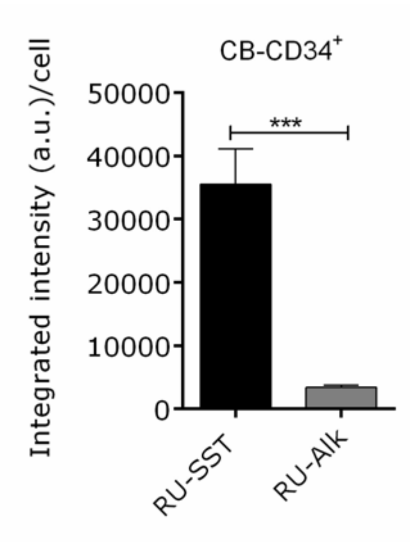

Supplemental Figure 8 A

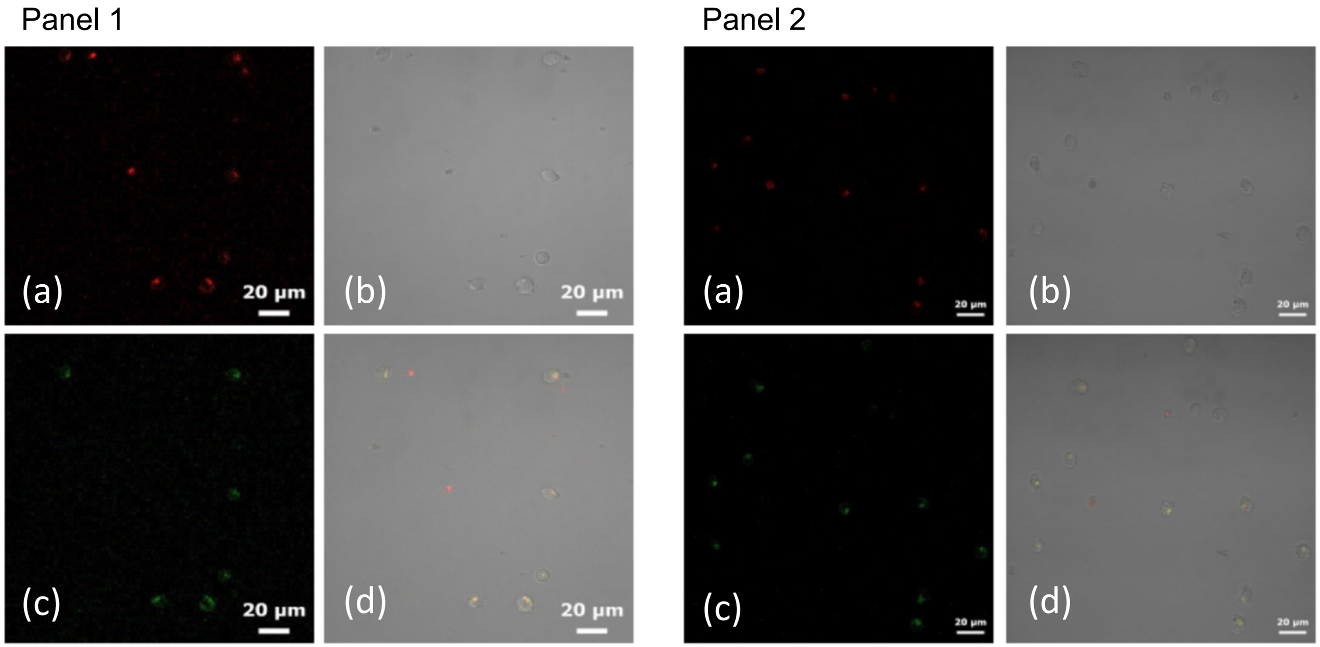

Supplemental Figure 8 B

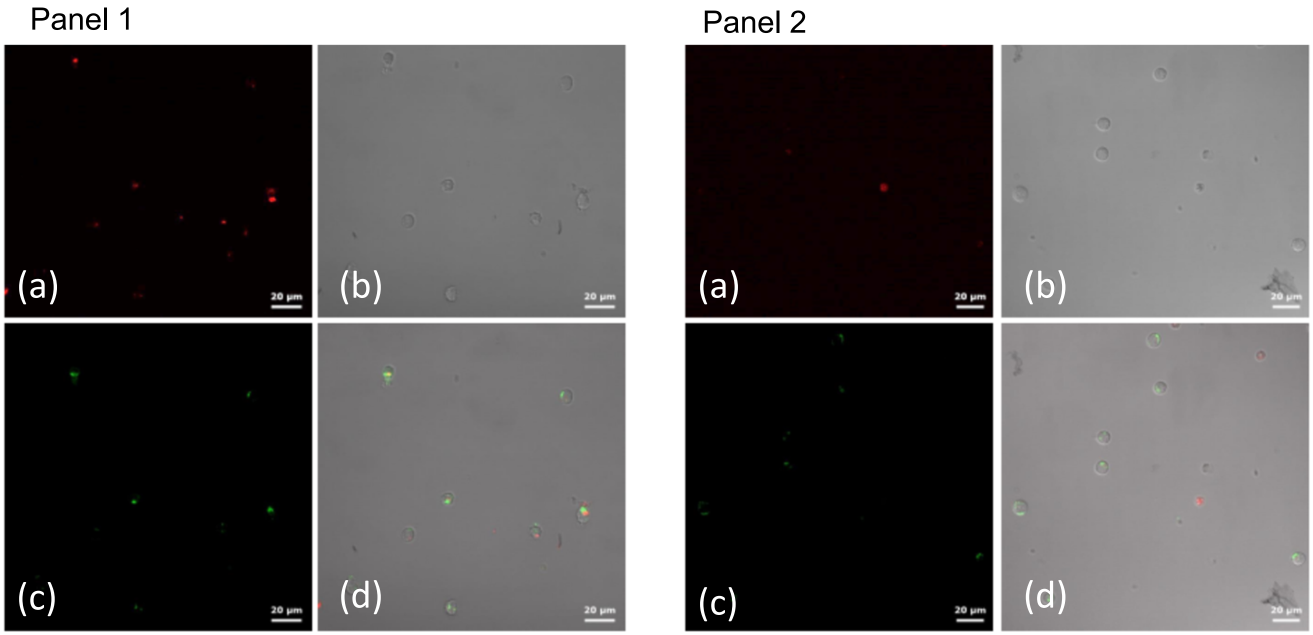

Supplemental Figure 8 C

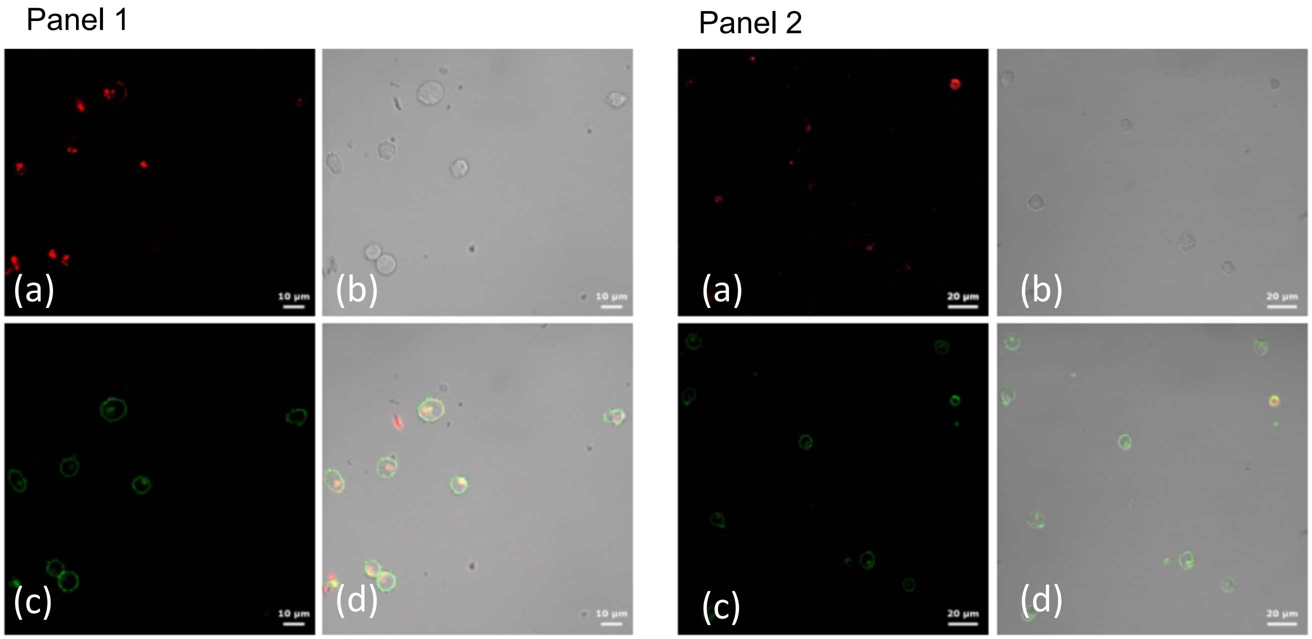

Supplemental Figure 8 D

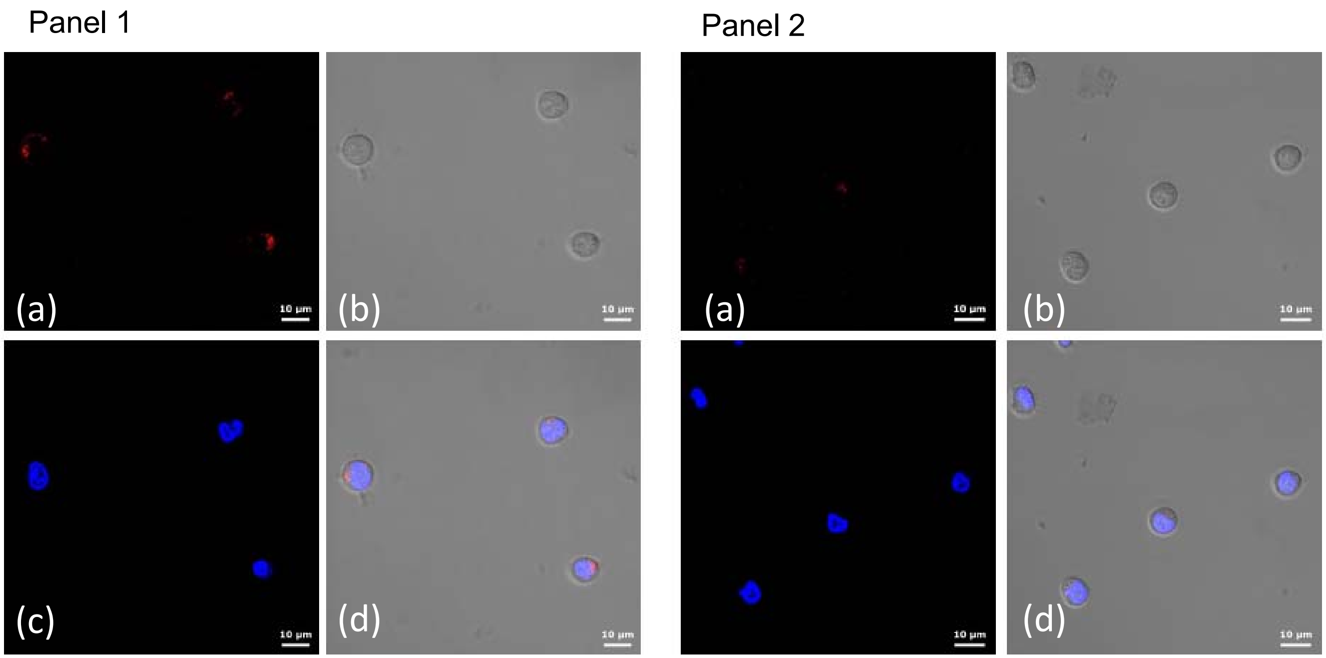

Supplemental Materials and Methods

Real time PCR

Total RNA from AML cells lines was isolated using the Direct-zol™ Kit (Zymo Research, Freiburg, Germany) and TRIzol®Reagent. cDNA preparation was performed using random hexamer primers (Primescript RT-PCR kit; TAKARA Clontech, Saint-Germain-en-Laye, France), as described by manufacturer´s protocol. The human TaqMan® SSTR2 expression probe (Hs00265624_s1) was purchased from ThermoFisher (Thermo Fisher; Darmstadt, Germany) and was assayed by TaqMan® quantitative real-time polymerase chain reaction (qRT-PCR).

Synthesis of Ruthenium-somatostatin (RU-SST) and Ruthenium Alkyne (RU-Alkyne) biohybrids

As SSTR2 was expressed on leukemic stem cell candidates, we generated an innovative biohybrid consisting of the tumor-associated peptide somatostatin and the photosensitizer ruthenium as previously described [1].RU-Alkyne was obtained by refluxing (bpy)2RuCl2 1 and bpy-alkyne 2 in ethanol/water 3: 1 for 3 h, followed by sephadex column chromatography purification affording RU-Alkyne in 45% yield. Subsequently, RU-Alkyne was conjugated to SST via Cu(I) catalysed cycloaddition in water with full conversion of SST. The RU-SST conjugate was isolated in 61% yield after HPLC purification (supplemental Figure 3A)

Stability study of RU-SST in fetal calf serum (FCS)

RU-SST was dissolved in 1 x PBS buffer with 10 % fetal calf serum (FCS) at a concentration of 49 µM and incubated at 37°C. 20 µL aliquots were taken at intervals of 0 h, 1 h, 2 h, 4 h and 8 h. Thereafter 180 µL of MeOH containing 5 ppm of Fmoc-

phenylalanine as an internal standard (Int. std.) was added to remove large proteins and the solution was centrifuged at 13.0 rpm for 30 min at 0°C. 150 µL of the supernatant was then pipetted for LCMS analysis. Experiments were performed in triplicates. Blank consisting of 20 µL water was also used as a negative control. LC-MS analysis was performed on a Shimadzu LC-MS 2020 equipped with an electrospray ionization source and a SPD-20A UV-Vis detector (Shimadzu, Duisburg, Germany). Aliquots (30 µL) were injected onto a Kinetex 2.6 µm EVO C18 100 Å, LC Column (50 x 2.1 mm). The column temperature was set at 40 ° C. The mobile phase consisted of 0.1% formic acid in water (B) and 0.1% formic acid in acetonitrile (A). The mobile phase was held for 1 min at 95% B and changed to a linear gradient from 95% to 30% B from 1 to 12 min, 30 to 5 % B from 12 to 13 min and hold at 5 % B for 3 min. The composition was changed back to 95 % over 0.01 min and hold for 4 min to for re-equilibration. The mobile phase flow rate was 0.4 mL/min. Identification of RU-SST and hydrolysis products were performed simultaneously by UV-VIS detection at 254 nm and selective ion monitoring (SIM) of the [M + H] + at m/z = 883, 663, 530 (RU- SST); 410 (Int. std). The amount of RU-SST in each sample was determined as a ratio of the integration of the mass chromatogram of RU-SST to the int. std. (supplemental Figure 3B-C). The data were plotted as mean ± SD.

Determination of the IC_{50}

For this purpose, we chose a high SSTR2 expressing cell line, HL60 [2]. Cells were treated with increasing amounts of both compounds RU-SST and RU-Alkyne from 10µM – 100µM. Thereafter, cells were washed and exposed to a 470 nm LED array with P = 23 ± 3 mW for 6 min (6.9 ± 0.9 J cm-2). The dark controls (not exposed to light) were performed in parallel. The cell viability was quantified by trypan blue exclusion. IC50 values were calculated using R-statistical package. Log concentrations were plotted against the probit values of ratio of dead cells. A linear regression model was fitted to find the relationship between the concentration and probit values. The IC_{50} was then estimated from the generated model by extrapolating the 50% probit value to the drug concentration.

Uptake studies

5000 cells of each of OCI-AML3 and CB were incubated with RU-SST (50 µM) and RU-Alkyne (100 µM) compounds for 4 hours, washed and were stained with Alexa Flour 430 and mounted on slides and measured for the fluorescence under confocal microscope. The live cell imaging was performed using a LSM 710 laser scanning confocal microscope system (Zeiss, Germany) coupled to an XL-LSM 710 S incubator and equipped with a 63x oil immersion objective. The emission of the RU-complex was recorded using a 530-710 nm filter and a 458 nm Argon laser for excitation. The acquired images were processed with ZEN 2011 software. The cellular uptake of RU-SST into human AML cells was investigated, since these cells express SSTR2 on the cellular surface. Equal quantities of RU-Alkyne and RU-SST were added to the culture medium of OA3 cells. After incubation for 4 h, the cells were washed to remove any conjugates that were not taken up and the cells were studied by laser scanning confocal microscopy. Laser excitation at 458 nm was applied that corresponds to the MLCT absorbance of the metal complex. The emission images were recorded in the range of 580–707 nm. Cells were stained with various subcellular organelle dyes with selectivity for membranes (Cell Mask Deep Red Plasma Membrane Stain), the nucleus (Hoechst 33342 Solution), mitochondria (Mito Lite Blue FX490, and lysosomes (Lyso Tracker Green DND-26).

Octreotid studies

In order to test the efficacy of a somatostatin analogue on AML cell lines, $5X10^4$ to $1x10^5$ cells from the OCI-AML, HL60 and THP-1 cell lines were incubated with increasing concentrations of Octreotid (Octreotid Bendalis GmBH, 82041, Oberhaching, Germany) ranging from 100nM to 10µM. Cell counts were measured by trypan blue exclusion 24, 48 and 72 hours after start of the incubation.

1 Wang, T. *et al.* Receptor selective ruthenium-somatostatin photosensitizer for cancer targeted photodynamic applications. *Chem Commun (Camb)* **51**, 12552-12555, doi:10.1039/c5cc03473f (2015).

2 Teijeiro, R. *et al.* Activation of human somatostatin receptor 2 promotes apoptosis through a mechanism that is independent from induction of p53. *Cell Physiol Biochem* **12**, 31-38, doi:10.1159/000047824 (2002).

Supplemental Figure Legends:

Suppl. Figure 1: Expression of SSTR2 on leukemic cell lines. q-PCR analysis of expression for SSTR2 was measured in AML cell lines belonging to various karyotypes (n=3). Bar graphs indicate the fold expression of SSTR2 compared to housekeeping gene TBP.

Suppl. Figure 2 A: Expression of Somatostatin Receptors (SSTR) in AML: Somatostatin receptor 1-5 (SSTR) expression (FPKM) was extracted from previously published RNA-Seq data sets of AML samples (n=43), which are deposited under accession number GSE49642.

Suppl. Figure 2 B: Microarray analysis from previously published datasets of AML patient samples from GEO profiles (GSE13159 , GSE15434 , GSE61804 , GSE14468 , The Cancer Genome Atlas TCGA). In comparison, microarray data from healthy bone marrow cells were analyzed (GSE42519).

Suppl. Figure 2 C and D: Expression of SSTR2 derived from RNA-Seq analyses of cord blood subpopulations from GEO profile (GSE 76234). C) The $\log_2$ expression is represented as colored circles (based on expression) and shown as hierarchical tree demonstrating the relationship between the subpopulations. D) Bar graphs indicate the mean RPKM values of SSTR 1-5 expression in human primary fetal and adult $CD34^+$ derived hematopoietic stem/progenitor cells (HSPCs) and erythroid progenitor cells from GEO profile (GSE 74053).

Suppl. Figure 3: Chemical structure of the biohybrid: A) The chemical structure of the biohybrid consisting of the peptide hormone Somatostatin conjugated to the

photosensitizer Ruthenium (RU-SST) and the control consisting of Ruthenium conjugated to alkyne RU-Alkyne (RU-Alk). Stability study for RU-SST: B) The calculation of the ratio of integration of mass chromatogram of RU-SST compared to the internal standard by LC-MS showed that the amount of RU-SST present in FCS remains consistent for up to 8h. C) Liquid chromatogram peaks show the absence of additional peaks at 254nm indicating the peptide fragmentation.

Suppl. Figure 4: Cell proliferation upon incubation with the somatostatin analogue octreotide: The graphs indicate the effect of Octreotide in AML cell lines OA3, HL60 and THP1 with increasing concentrations of 100nm, 1µm and 10µm. Cell proliferation was determined by trypan blue exclusion after 24, 48 and 72 hrs

Suppl. Figure 5: Detection of apoptosis by Annexin V staining: OCI-AML3, THP1 and MM6 cell lines were incubated with RU-SST and performed Annexi V staining as described in Materials and Methods. Bar graphs indicate the mean ± SEM of percent live cells after RU-SST treatment (n=3). Significance was calculated by using 2-way ANOVA. P-values designated as (* $p<0.05$; ** $p<0.01$; *** $p<0.001$; **** $p<0.0001$).

Suppl. Figure 6: Expression of SSTR2 in primary AML samples: SSTR2 expression was determined by qRT-PCR in AML patients (n=13) and $CD34^+$ CB cells (n=5). Expression is shown as delta Ct value compared to the house keeping gene TBP. Note: the lower the value the higher the expression. AML samples that were tested for their clonogenic potential in CFC assays are represented as colored circles. The patient characteristics are summarized in Table 1.

Suppl. Figure 7 A and B: Uptake studies of ruthenium complexes in normal $CD34^+$ cells: A) Confocal images of RU-SST (50µm) and RU-Alkyne (100µm) incubated with $CD34^+$ enriched cells from cord blood for 4 hrs. The laser intensity was

measured for Alexaflour (430nm), a FITC channel in the confocal microscope. Uptake characteristics demonstrate a high uptake of RU-SST compared to the unconjugated RU-Alkyne in $CD34^+$ CB cells. B) Bar graphs indicate the average integrated intensity per cell in both experimental arms. Values mentioned are mean ± SEM (RU-SST n=7; RU-Alk n=8). P-values shown as (*** p<0.001).

Suppl. Fig. 8 A-D: Localization studies of ruthenium complexes in normal $CD34^+$ cells: Confocal microscopy images of $CD34^+$ CB cells incubated with RU-SST (Panel 1) and RU-Alkyne (Panel 2) and treated with organelle trackers for A) lysosomes, B) mitochondria, C) membrane and D) nuclei. The panels of each figure show (a) RU-SST emission, (b) corresponding bright field images, (c) emission from the organelle trackers, and (d) overlay of all three images.
